# Bovine leukosis virus, bovine viral diarrhea, and bovine neosporosis seroprevalence in specialized dairy herds in Antioquia-Colombia

**DOI:** 10.1007/s11250-023-03685-2

**Published:** 2023-09-01

**Authors:** Cristian C. Rúa Giraldo, Albeiro López Herrera, Tatiana Ruiz-Cortés

**Affiliations:** 1 Master Student in Animal Sciences, Universidad Nacional de Colombia Medellín Campus, Medellín, Colombia; 2 Facultad de Ciencias Agrarias, Research Group Biogénesis, Universidad de Antioquia, Medellín, Colombia; 3 Sede Medellín, Facultad de Ciencias Agrarias, Research Group Biodiversidad Y Genética Molecular “BIOGEM”, Universidad Nacional de Colombia, Medellín, Colombia

**Keywords:** Antibodies, Dairy herds, ELISA, Epidemiology, Milk-producing municipalities, Variability

## Abstract

Enzootic bovine leukosis (EBL) is a chronic infectious disease caused by the bovine leukosis virus (BLV), a *Deltaretrovirus*. Bovine viral diarrhea (BVD) is an infectious disease caused by a pestivirus. Bovine neosporosis is caused by the obligate intracellular parasite *Neospora caninum* (Nc). These pathogens can have horizontal (postnatal) or vertical (transplacental) transmissions and affect the productive and reproductive performance of infected bovines. This work aimed to detect BLV, BVD, and Nc seroprevalence in specialized dairy cattle from the north, east, and Aburrá Valley regions of the Department of Antioquia, the highest in milk production regions in Antioquia. A total of 599 blood samples, obtained from 53 specialized dairy cattle herds, were evaluated by the ELISA test. The results revealed a seroprevalence of 41.13% for BLV (242/599), 28.48% (163/599) for Nc, and 22.7% (132/599) for BVD. Regarding the regional seroprevalence evaluation, BLV was found in 47.02% of the samples from the east, 36.87% from the north, and 46.02% from the Aburrá Valley. Nc was found in 31.03% of the samples from the east, 24.26% from the north, and 36.63% from Aburrá Valley. BVD was found in 21.62% of the samples from the east, 25.03% from the north region, and 10.39% of the samples from the Aburrá Valley. It is highlighted by these results that the north region, with the highest milk production in Antioquia, had the lowest BLV and Nc seroprevalences but the highest seroprevalence of BVD. BLV has increased in Antioquia in recent years, and as an immunosuppressive infection, opportunities for other pathogens are created by it. A significant statistical difference was found in the average prevalence of the pathogens according to the municipality, cattle breed, and region of origin of the sample. The seroprevalence of these pathogens in specialized dairy herds in Antioquia can be classified as medium-low. However, it is recommended that biosecurity practices should be maximized to avoid the spread of these pathogens due to the variability detected in the region, municipality, breed group, and herd age. The rapid and efficient diagnosis of these three pathogens through reliable methodologies will allow for the control of dissemination in dairy herds.

## Introduction

Specialized dairy systems located in the Department of Antioquia (Colombia) are of direct and indirect relevance in the economies of the regions where they are located. Thus, strategies that favor their progress must be constantly focused on, as the well-being of producers depends on specialized dairy systems (FEDEGAN 2018). Enzootic bovine leukosis (EBL) is a disease with high morbidity and low mortality rates. This disease is caused by infection by the bovine leukosis virus (BLV) (De la Sota 2005). EBL mainly affects cattle, especially those dedicated to milk production, by infecting B lymphocytes and generating persistent lymphocytosis (PL) in cows. As a result, BLV-infected animals become immunosuppressed, and economic losses are derived from the decreased milk production of such individuals compared to uninfected animals. Reducing the immune response capacity is estimated to allow other pathogens, such as bovine viral diarrhea (BVD) and *Neospora caninum* (Nc), to infect cattle. Ultimately, these other pathogens will cause health problems that will eventually worsen adverse effects on production and reproduction indices (Emanuelsson et al. 1992; De la Sota 2005; Ortega et al. 2016).

BLV, a member of the genus *Deltaretrovirus* (Retroviridae family), causes EBL, which affects the cells of the lymphoid lineage, specifically B lymphocytes (Beyer et al. 2002; Wu et al. 2003). Most infected animals (~ 60%) do not show clinical or hematological signs and become asymptomatic carriers of the virus. Approximately 30–70% of infected animals may present a sustained increase in the absolute number of lymphocytes in the bloodstream as permanent lymphocytosis (PL), and 0.1–10% of bovines with more than 3 years of infection suffer from some lymphosarcoma (LS) (Dees et al. 1996; WOAH 2021). The detection of BLV-infected cattle is essential for infection control since most animals do not show clinical sign, which makes their detection difficult through routine examination practices. However, some animals show exophthalmia, a degeneration of the retro-ocular tissue or of the internal structures of the eye, which is a sign of the disease (Malatestinic 2003). In other cases, the lymph nodes enlarge in animals with more than 3 years of infection and are detected on palpation. The main serological methods used to identify BLV-infected cattle are agar gel immunodiffusion (AGID) and enzyme-linked immunoassay (ELISA) (Martin et al. 2001). Both diagnostic techniques are recommended by the World Organization for Animal Health (2021) for detecting specific antibodies against the gp51 and p24 proteins that are part of the virus envelope.

BVD belongs to the genus *Pestivirus* (Flaviviridae family) (Donis 1995). The primary source of infection and reservoir of the virus are persistently infected cattle. Most of the animals are infected in their early embryonic periods. They are born clinically healthy but continuously shed large amounts of the virus throughout their lives in the nasal secretion, saliva, urine, fecal matter, tears, semen, and milk and are called persistent infected (PI) animals (Navarro et al. 2023). Acutely infected animals are also a source of infection, although they shed the virus in low amounts and for short periods (Houe 1999). Most BVD infections are subclinical or moderate, with high morbidity and low mortality rates (Baker 1987; Kelling 1996). One of the signs of acute infection is bovine neonatal diarrhea complex when the passive transfer of antibodies from the cow to the newborn calf fails. Reports of severe acute infection with high morbidity and mortality associated with highly pathogenic viruses, characterized by high fever, respiratory signs, diarrhea, abortions, drop in milk production, and sudden death, are becoming more frequent (Drake et al. 1996). The most significant economic impact of BVD infection is caused by reproductive disorders (Moennig and Liess 1995). Serological tests, such as ELISA, neutralization, and seroneutralization, have been suggested as suitable tools for detecting the presence of BVD in bovine populations. Specifically, neutralization and seroneutralization tests can detect neutralizing antibodies against the virus that appear in the serum of post-infected animals (OIE 2010).

Neosporosis is a pathology with worldwide diffusion that has been reported as one of the leading causes of losses due to abortion in bovines. It is caused by the protozoan *N. caninum* (Nc) (Dubey 1999). Dogs are definitive and intermediate hosts of these protozoans. Dogs eliminate oocysts in their feces, which are ingested by other intermediate hosts, such as cattle, by consuming contaminated pastures or feed. Once Nc infects a cattle population, the main spreading route to maintain the infection is the transplacental infection (Lindsay et al. 1998; McAllister et al. 1998). Asymptomatic congenital infection predominates in adult bovines, potentially causing various effects, such as abortion and mummification, and, more rarely, neurological signs in neonates. Abortions occur more frequently between four and six months of gestation, and autolysis occurs very frequently in the fetus (Easton et al. 2003). Nc infection may be detected using histopathological, molecular, isolation techniques, and immunodiagnostic tests, such as ELISA, which is considered a stand-out test (Dubey 2003).

**Image 1:**
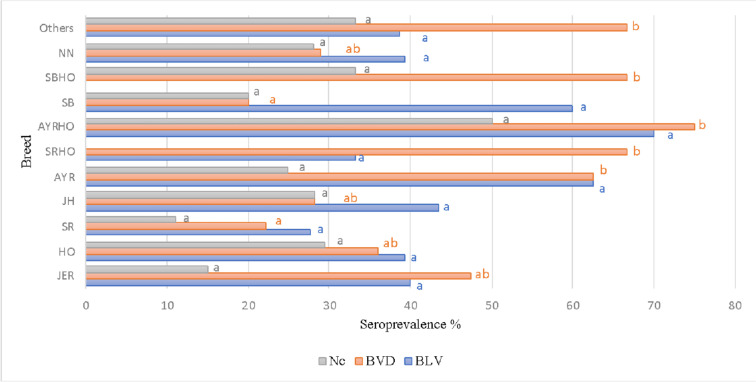
Geographical location of the study regions in the department of Antioquia

This research determined the seroprevalence of BLV, BVD, and Nc in specialized dairy herds in Antioquia-Colombia and compared the seropositivity between regions, municipalities, and bovine breeds (Image 1).

## Material and methods

### Study population

The study population comprised 224,714 cows milked in specialized dairy herds in the Aburrá Valley, north, and east regions of the Department of Antioquia, Colombia, the largest milk production regions in this department (DANE 2017). The sample size was calculated with an expected prevalence to BLV of 44% (Úsuga-Monroy et al. 2018a, b), a relative error of 10% (an absolute error of 4.4%), and a design effect of 1.2, yielding a sampling universe of 599 bovines belonging to 53 herds. They represented several breeds and crosses, from animals aged a few months to 11 years. Cows belonging to the three milk production regions were sampled in 16 municipalities of the Department of Antioquia. Three municipalities were from the Aburrá Valley region (Bello, Girardota, and Medellín), seven were from the north region (Belmira, Don Matías, Entrerríos, San José de la Montaña, San Pedro, Santa Rosa, and Yarumal), and six were from the east region (Abejorral, Carmen de Viboral, La Ceja, Marinilla, Rionegro, and Santuario). The samples were distributed as follows: 33 herds were located in the north, 10 in the east, and 10 in the Aburrá Valley. Management, feeding, and health procedures varied and depended on each farm. These are located in regions ecologically classified as low montane very humid forest zones (bmh-mb), which have as general climatic limits an approximate temperature between 12 and 18 °C and an average annual rainfall between 2000 and 4000 mm. Normally they extend in an altimetric band from 1800 to 2800 m above sea level. A proportional number of samples was identified before sampling and randomly obtained in each herd, depending on the herd size. A blood sample was obtained (see below) from the following breeds used in the specialized dairy in Antioquia: Jersey (JER; *n* = 40), Holstein (HO; *n* = 191), Swedish Red (SR; *n* = 17), Swiss Brown × Holstein (SBHO; *n* = 4), Jersey × Holstein (JH; *n* = 111), Swedish Red × Holstein (SRH; *n* = 6), Ayrshire (AYR; *n* = 9), Ayrshire × Holstein (AYRHO; *n* = 4), Swiss Brown (SB; *n* = 4), unidentified breed (NN; *n* = 197), and others (Black-and-white (BON) × Holstein, Norman (NORM), Angus (ANG) × Holstein, SR × JER, BON × HO, NOR × HO, ANG × HO and JANG, and Gyrolando (GYR)) (*n* = 16). Therefore, the sample universe constituted a representative value of the information found in the specialized dairy from Antioquia-Colombia.

### Plasma and sera samples

Blood samples were taken from the middle coccygeal vein, with an 18-G needle in a vacuum vacutainer system (VACUETTE®) and EDTA as an anticoagulant to obtain plasma to determine BLV seropositivity. The samples were homogenized using the inversion method and transferred under refrigerated conditions (4 °C) to the Animal Biotechnology laboratory of the Universidad Nacional de Colombia, Medellín. The samples were centrifuged at 3,000 × *g* for 10 min, and the plasma was stored at – 20 °C until analysis.

The same protocol was followed for the diagnosis of seropositivity to BVD and Nc. Even so, tubes without anticoagulants were used to obtain blood sera and were transported at 4 °C to the Immunodiagnostic Laboratory of the University of Antioquia. Two 15 mL/cow tubes were centrifuged at 3,000 × *g* for 10 min at 10 °C. Sera were recovered and stored at – 20 °C until analysis.

### Sample processing

For the BLV seropositivity analysis, the plasma samples were processed using the ELISA technique, according to the manufacturer’s instructions (Svanovir® BLV gp51-Ab), in 96-well polystyrene plates with 100 μL of antigen dilution buffer per well. Four microliters of the positive and negative control sera and 4 μL of the plasma samples were seeded in duplicate in a total volume of 100 μL per well. The plate was sealed and incubated at 37 °C for 1 h. The plate was washed three times with Abcam’s phosphate-buffered saline (PBS)-Tween buffer, and 100 μL of horseradish peroxidase (HRP) conjugate was added per well, incubated for 1 h at 37 °C, and subsequently washed three times with Abcam’s phosphate-buffered saline (PBS)-Tween buffer. After washing, 100 μL of the substrate solution (ABTS) was placed in each well and incubated for 10 min at room temperature. The reaction was stopped by adding 50 μL of stopper solution to each well. The reading was conducted in a spectrophotometer with a filter at 450 nm, using a corrected optical density ratio (OD sample/OD-positive control) to interpret the results.

For the Nc analysis, the serum samples were processed using the ELISA technique according to the manufacturer’s instructions (IDEXX Neospora X2). In the antigen-coated polystyrene plates, 100 μL of undiluted negative control was added in duplicate to the wells, 100 μL of undiluted positive control was added to the wells, and 100 μL of each diluted sample was added to the wells (1:100 with the kit diluent). Subsequently, the samples were incubated for 30 min at 18–26 °C. Next, each well was washed with approximately 300 μL of washing solution four times. Then, 100 μL of the conjugate was added to each well, allowing it to incubate for 30 min at 18–26 °C. Later, each well was washed with approximately 300 μL of washing solution four times. Finally, 100 μL of TMB substrate (3,3’,5,5’-tetramethylbenzidine) was added to each well and incubated for 15 min at 18–26 °C. The reaction was stopped by adding 100 μL of the stopping solution in each well to obtain the absorbance values at 620–650 nm.

The serum samples were processed using the ELISA technique for the BVD analysis according to the manufacturer’s instructions (IDEXX BVDV p80 Ab). In the polystyrene plates lined with the antigen, 50 μL of buffer dilution was added to each well, 50 μL of negative control was added to two wells, 50 μL of positive control was added to one well, and 50 μL of each sample was added to the remaining wells (one well per sample). Each well content was homogenized using a microplate shaker, and the microplate was covered and incubated for 1 h at 18–26 °C. Next, each well was washed with approximately 300 μL of washing solution 3–5 times. Then, 100 μL of the diluted conjugate was added to each well, and the microplate was covered to incubate for 30 min at 18–26 °C. Each well was washed three times with approximately 300 μL of washing solution, and 100 μL of TMB substrate (3,3’,5,5’-tetramethylbenzidine) was added and incubated for 20 min at 18–26 °C in a dark place. The reaction was stopped by adding 100 μL of stop solution to each well. Finally, the OD of the samples and controls was measured at 450 nm.

According to the OD, those samples that obtained a percentage positive value greater than or equal to 10 were considered positive for the presence of antibodies against the BLV protein (gp51), according to the manufacturer’s criteria (yellow color). For BVD, those samples that obtained a biological reference percentage value lower than or equal to 40 were considered positive for the presence of antibodies against the protein, and those samples that obtained a biological reference percentage value greater than 40 and less than 50 were considered doubtful. For Nc, samples that obtained a biological reference value greater than or equal to 0.50 were considered positive for the presence of antibodies.

### Statistical analysis

The percentage of general seroprevalence of each pathogen and the positivity percentage of each pathogen for each age group, breed, municipality, and region were determined in specialized dairies in Antioquia. The animals were grouped according to the region of origin of the sample. The data supplied by the Colanta dairy cooperative and those resulting from the information collected during sample processing were integrated into a single Excel database to obtain reliable information for the analyses. For BLV, BVD, and Nc, chi-square (*χ*^2^) tests were performed since there was no normal distribution in the results. Comparisons between the averages of the observed prevalence frequencies of the pathogens between age groups, breeds, municipalities, and regions of origin of the sample were performed. The statistical package R version i386 4.2.0 (R Core Development Team 2023) was used. The significance value was assumed when *p* was lower than 0.05.

## Results and discussion

BLV is distributed worldwide, and its prevalence varies widely between countries and continents. BLV has a high seroprevalence in the USA, one of the leading producers of bovine milk (83.9% of worldwide production; USDA 2007). High bovine seroprevalences have also been found in South America. For instance, seroprevalences were approximately 24% in Chile (Grau and Monti 2010), 27.78% in Brazil (Bianchi 2003), and 27.78% in Uruguay (Algorta 2014).

The results of general seropositivity and region prevalence for the three pathogens evaluated are shown in Table 1. In this study, the seroprevalence for BLV in the specialized dairy in Antioquia was 41.13% (242 positive animals out of 599 sampled), demonstrating the circulation of the bovine leukosis virus in the dairy herds of the department. Bautista et al. (2013) found a seroprevalence of 15% in serum samples from the Department of Boyacá obtained from heifers and cows in production. The lower value found could be explained by the type of production system since these authors evaluated the seroprevalence of the virus in double-purpose cattle and not in specialized dairy cows. In another study conducted in Colombia, a seroprevalence of 54.6% was reported in 1000 samples of pure Holstein bovines from 10 dairy municipalities in Antioquia (Úsuga-Monroy et al. 2018a, b). It should be noted that the lower seroprevalence found in our study may be due to the presence of different breeds and crosses that are used in specialized commercial dairy herds in Antioquia.

**Table 1:** Seroprevalence of BLV, BVD, and Nc in three regions of the Department of Antioquia-Colombia

Region	North	East	Aburrá Valley	Total
Number of animals	393	89	117	599
BLV +	145	42	55	242
BLV −	248	47	62	357
Seropositives (%)	**36.87**^**a**^	**47.02**^**b**^	**46.02**^**b**^	**41.13**
BVD +	104	16	12	132
BVD −	289	47	105	441
Seropositives (%)	26.47^a^	**17.54**^**ab**^	**9.8**^**b**^	**22.7**
Nc +	89	30	44	163
Nc −	304	59	62	425
Seropositives (%)	**22.67**^**a**^	**33.89**^**a**^	**37.28**^**a**^	**28.48**

Different letters depict statistical differences (*p* < 0.05)

Significant statistical differences are highlighted in black

In this investigation, a seroprevalence of 28.48% was found in specialized dairies in Antioquia for Nc and 22.7% for BVD. These results differ from those reported in a study of cattle from an indigenous reservation in Cauca (Colombia), where the infection with the highest seroprevalence of 50% was for BVD, followed by bovine neosporosis (36%) (Valdez et al. 2018). Likewise, high seropositivity for bovine neosporosis (63%) was found in the Department of Santander, followed by BVD (29.7%), and leukosis (21.8%) in a population of 440 cows (Vargas-Niño et al. 2018).

A statistical significance was found between the means for the north region and the regions of Aburrá Valley and the east in this investigation. The highest BLV seroprevalence was found in the east, with a percentage of 47.02%, followed by Aburrá Valley, with a 46.02% seropositivity. Finally, the north had a seropositivity of 36.87%.

No statistically significant differences were found between the means of Nc for the three regions. Specifically, the highest seroprevalence was found in the Aburrá Valley, with a percentage of 36.63%, followed by the eastern region, with 31.03% seropositivity, and finally, the north region, with 24.26%.

A statistically significant difference was found between the mean of the north and the Aburrá Valley regarding BVD. Still, no statistically significant differences were found among the regions. The highest seroprevalence was found in the north (25.03%), followed by the east (21.62%), and by the Aburrá Valley (10.39%).

At the municipal level, the highest BLV seroprevalence was found in the herds in La Ceja (east region; 57.87%). The lowest seroprevalence was found in the herds from San José de la Montaña (north region; 19.44%). For Nc, the highest seroprevalence was found in the herds in Bello (Aburrá Valley; 55.55%). The lowest seroprevalence was found in the herds in Abejorral (east; 0%). Finally, the highest BVD seroprevalence was found in the herds in San Pedro de los Milagros (north; 37.54%), and the lowest seroprevalence was found in the herds in Abejorral (east; 0%). These values showed the wide pathogen distribution in the different municipalities in the leading specialized dairy herds in the Department of Antioquia. Statistical significance was found when comparing the BVD frequencies (*χ*^2^ = 13.58; *p* = 0.009) and the Nc presence (*χ*^2^ = 16.70; *p* = 0.002) at the regional level. Still, at the regional level, a statistically significant difference was not found for BLV seroprevalences (*χ*^2^ = 5.83; *p* = 0.21). Additionally, there was no statistical significance for BLV at the municipality level (*χ*^2^ = 16.40; *p* = 0.36). Nonetheless, there were differences in BVD prevalence at the municipality level (*χ*^2^ = 113.80; *p* = 2.99^−17^) and Nc seroprevalences at the municipality level (*χ*^2^ = 28.53; *p* = 0.02).

Multiple results have been published related to the presence of BLV in different Colombian departments. A seroprevalence of 21% was found in animals presenting reproductive problems in the Department of Córdoba (Betancur and Rodas 2008), a lower value than that found here. This may be due to the system production type since it was evaluated in Zebu and double-purpose cattle and not in specialized dairy systems. The serological prevalence of BLV in specialized dairy herds in the Municipality of San Pedro de los Milagros in the Department of Antioquia was 12.07%, using the AGID technique. Specifically, a high prevalence was found in the age group from 5 to 9 years (Aguilar 1989). The same technique was used to evaluate blood serum samples from the Sabana de Bogotá and the Ubaté and Chiquinquirá Valleys, and seroprevalence of 45.28% was found (Alfonso et al. 1998).

The results refer to the detection of BLV in bovine plasma samples and BVD and Nc in bovine serum. There was no statistical significance between the mean frequencies related to the presence of BLV in different breeds (*χ*^2^ = 16.69; *p* = 0.34). This coincides with the study reported by Betancur and Rodas (2008), who found no statistical significance between the prevalence of the diseases in specialized dairy breeds. No statistical significance was found in our study for the presence of Nc in different breeds (*χ*^2^ = 14.62; *p* = 0.48). Still, there was a statistical significance between the presence of BVD in different breeds (*χ*^2^ = 29.19; *p* = 0.02): Brown Swiss (BS) and Swedish Red (SR) breeds presented more seropositivity than BSHO, AYRHO, SRHO, and AYR breeds.

**Fig. 1:**
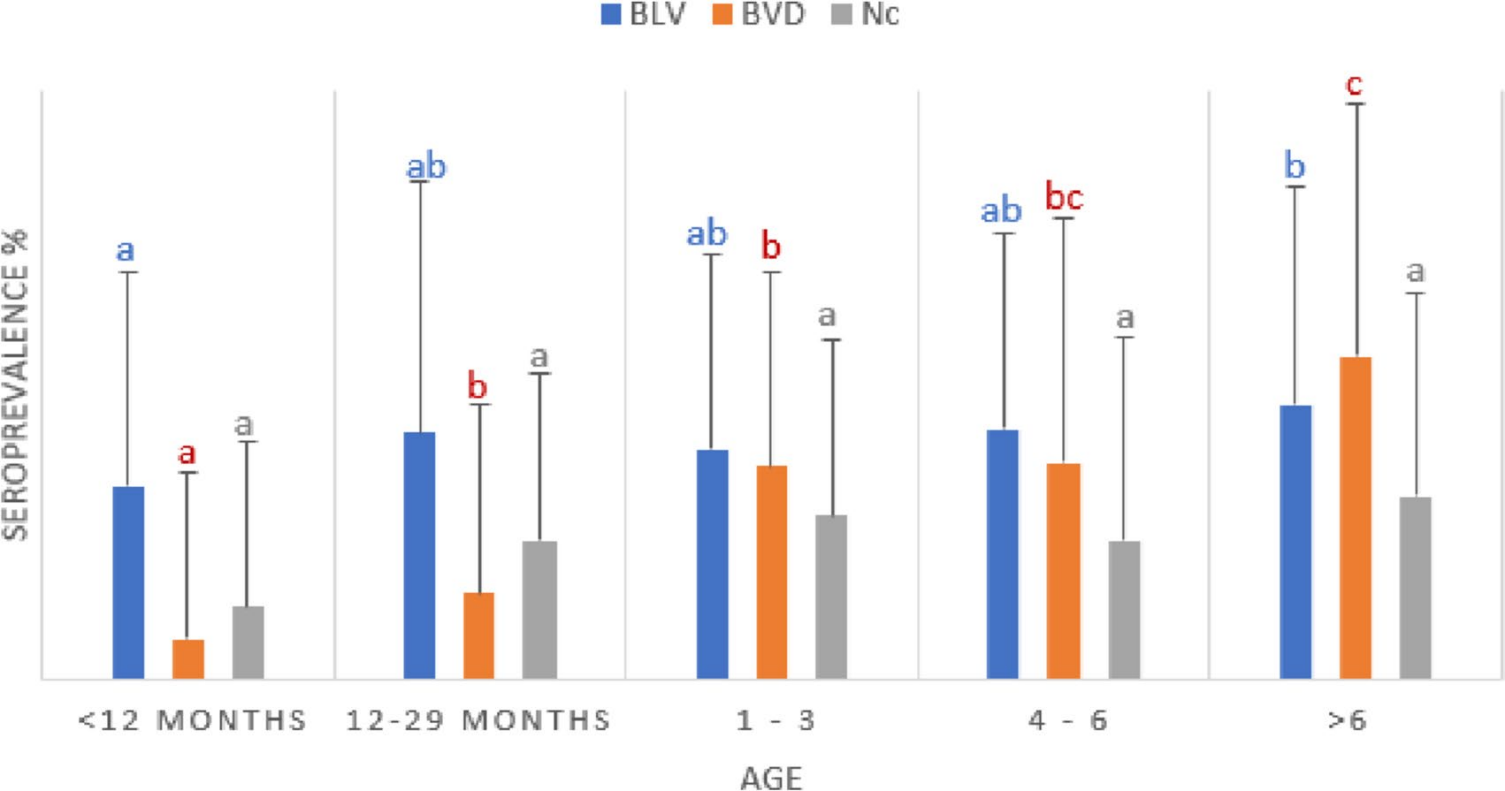
Seroprevalence of BLV, BVD, and Nc in the main breeds used in specialized dairy production in Antioquia (Colombia). Different letters depict statistical differences (*p* < 0.05). JER Jersey, HO Holstein, SR Swedish Red, JH Jersey × Holstein, AYR Ayrshire, SRHO Swedish Red × Holstein, AYRHO Ayrshire × Holstein, SB Swedish Brown, SBHO Swedish Brown × Holstein, NN unidentified breed, Other Other breeds, Nc *Neospora caninum*, BVD bovine viral diarrhea, BLV bovine leukosis virus

As observed in Fig. 1, the percentage of BLV prevalence was high in the AYRHO and AYR breeds (100% and 62.5%, respectively). The BS and SRHO breeds had the lowest BLV presence among the sampled breeds (27.77% and 33.33%, respectively), except for breeds with 0% seroprevalence, such as BSHO, BONHO, NORHO, ANGHO, BON, and JANG. The percentage of BVD positivity was high in the BON and JANG breeds (100% of both). The RS and JH breeds had the lowest BVD presence (22.22% and 28.3%, respectively), except for the sampled breeds in which BVD was not found (i.e., NORHO and ANGHO). The positivity of Nc in the sampled breeds was high in the BONHO and TRI individuals (50% and 40%, respectively). Individuals in the JER and RS breeds had the lowest positivity (15% and 11.11%, respectively). The breeds in which BVD was not found were RSHO, NORHO, and ANGHO. The presence of these pathogens in dairy cattle breeds is more significant than in other bovine production systems (Chamizo 2005). This is because there is extensive exposure to inadequate intensive management practices, such as reusing needles during vaccination processes (Ortega et al. 2016). For instance, reusing contaminated gloves on different animals during the palpation exam leads to the spreading of the virus (Divers et al. 1995). These practices explain the differences between the seroprevalences in this work and those reported by other authors.

The BLV seroprevalence for HO in our study was 39.34%. In a study conducted in Valle del Cauca, it was 83.3% (Hernández et al. 2011, like another study in 2012, with 77.8% positivity for HO cows in Valle del Cauca (Cadavid 2012). These subregions and municipalities are highly related to the presence of the virus. The association between the region and the presence of the disease has also been established elsewhere (Hernández et al. 2011). It was discussed that this association might be related to the place of origin and differences in production systems. The seroprevalence of BLV in the Department of Antioquia during 2004 was 44% in HO dairy cattle, with variations within farms ranging from 21 to 85% (FEDEGAN 2015). At the same time, variations among municipalities analyzed with the PCR diagnostic method were between 16 and 88% but showed similar molecular prevalence (44%).

It needs to be considered that EBL is typically a chronic disease. It is pertinent to epidemiologically assess whether there is a statistical significance between the prevalence of the disease in animals of different ages and parturition number. For BLV and BVD, a statistically significant difference was found between the means of seroprevalence of animals younger than 12 months and animals with more than 6 years. It was also found that there was a statistically significant difference between the BVD seroprevalence in animals younger than 12 months, animals between 12 and 29 months, animals with 1–3 and parturitions, and animals with 4–6 parturitions. No statistically significant differences were found for Nc regarding the means related to age or number of parturitions in the animals.

**Fig. 2:**
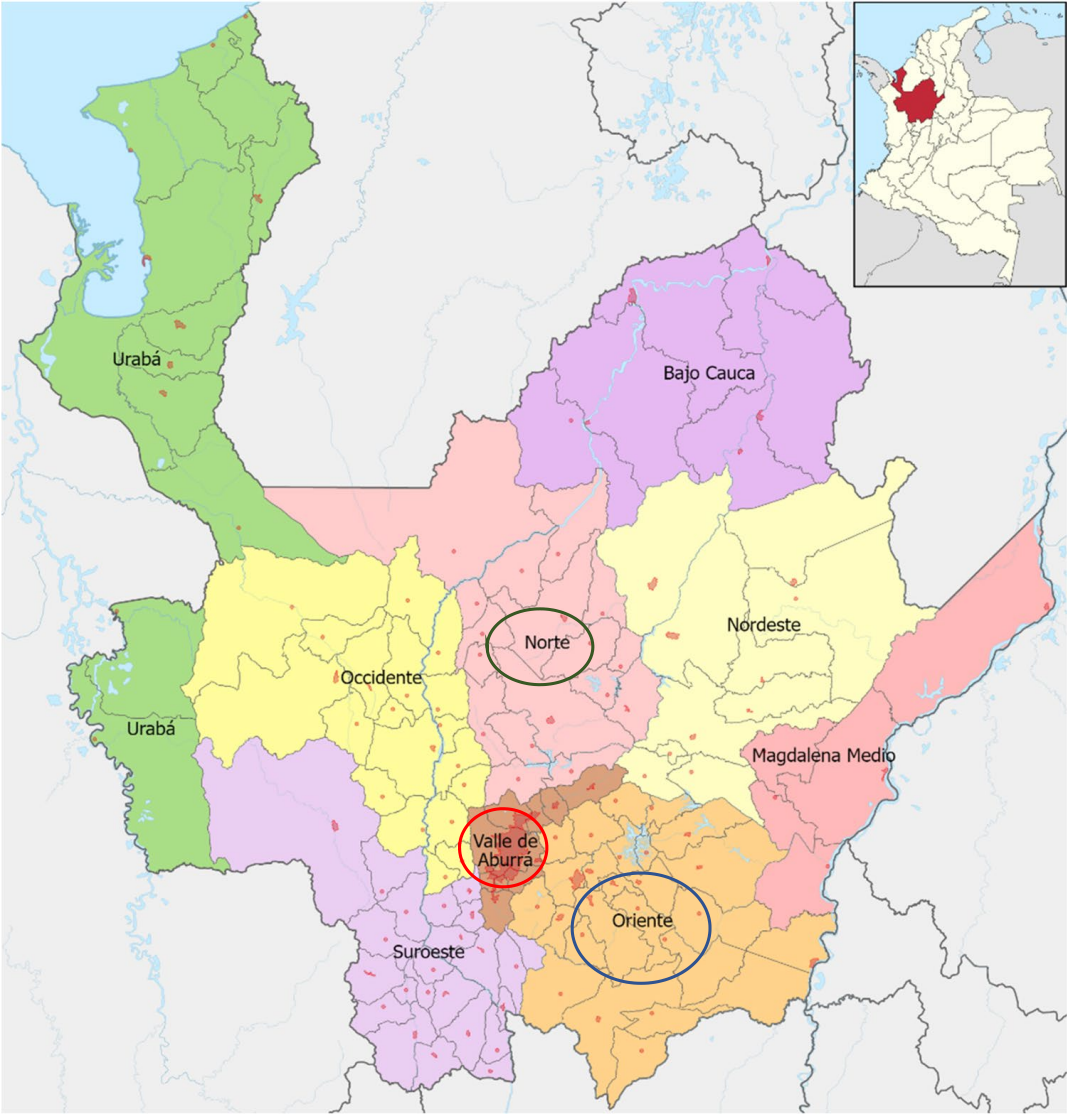
Seroprevalence of BLV, BVD, and Nc according to age (months) and parturition number (1–3, 4–6, and more than six births) in specialized dairy cows from the Department of Antioquia (Colombia). Different letters depict statistical differences (*p* < 0.05)

As shown in Fig. 2, there was 32.88% seropositivity to BLV, 6.85% seropositivity to BVD, and 12.33% seropositivity to Nc when the analysis was conducted in individuals > 1 year of age. There was a 41.82% seropositivity to BLV, 14.55% seropositivity to BVD, and 23.64% seropositivity to Nc in individuals aged 1 year and that had their first birth. There was 38.95% seropositivity to BLV, 36.14% seropositivity to BVD, and 27.71% seropositivity to Nc in individuals with 1–3 births. There was 42.45% seropositivity to BLV, 36.79% seropositivity to BVD, and 23.58% seropositivity to Nc for individuals with 4–6 births. There was 46.47% seropositivity to BLV, 54.92% seropositivity to BVD, and 30.98% seropositivity to Nc in individuals with more than six births. Significant seropositivity to BLV and BVD was found in individuals with more than three births, in agreement with the findings of Betancur and Rodas (2008), Nava et al. (2011), and Ohno et al. (2015). These researchers reported that the highest seropositivity was found in animals with more than three parturitions.

Gutiérrez et al. (2011) also found that the BLV and BVD seroprevalences varied between 40 and 60% in animals that had more than three calving. These values coincide with those found in the present study, which varied between 36.79 and 54.92%. Still, the seropositivity values found by Gutiérrez et al. (2011) in newborn animals until the first parturition differed from those found in this study, which varied between 11 and 24% and was lower than range of variation of 6.85% and 41.82% found in our research. This was consistent with the study reported by Resoagli et al. (1991), who indicated that BLV occurs more frequently in adult animals. Although these researchers found some cases occurring at young ages, the disease only manifested during adulthood and could also be explained by accumulated exposure to infected animals over the years. In this sense, it is clear that infected and uninfected animals were in the same environment and under the same maintenance conditions. Therefore, it can be said that the risk factors that predispose animals to the disease due to the effect of these pathogens can be given mainly by the breed, their handling, and the practices conducted in the herds. This latter aspect must be considered to prevent disease manifestations and control their spreading. Acceptable livestock practices must be encouraged in the place of study and all dairy production regions in the country.

There are various factors as potential routes of infection, these are related to deficiencies in zootechnical and veterinary control in specialized dairy herds, such as ignoring the health status of animals and transferring animals within and between herds, which are the main causes of dissemination. of pathogens. In addition, the lack of adequate controls from relevant authorities and the lack of awareness among producers are significant issues enhancing pathogen dissemination. This lack of efficient controls resulted in infected animals being introduced in regions and herds that were once pathogen-free (Grau and Monti 2010). According to Castelli et al. (2001), the type of livestock production system influences disease prevalence. Animals in intensive farms are exposed to overcrowding, high contact with other animals, and several handling by operators. Such features are typical in specialized dairies in high tropical regions. Poor management and veterinary practices within each herd also caused seropositive animals to transmit the disease to uninfected animals within the herds (Grau and Monti 2010). The most frequent veterinary misconducts include using the same needle to vaccinate several animals, deficient programs for hematophagous parasite control (Delgado et al. 2009), diagnostic palpations and artificial insemination conducted without adequate aseptic practices, and the reuse of palpation gloves.

The differences between the BVD seroprevalence found in this study concerning other authors may be due to sampling size and other factors, such as the region or municipalities (Rivera et al. 2018). Similarly, age, vaccination, abortion history of cows, and history of diarrhea are associated with exposure to the BVD virus (Buitrago et al. 2018). In contrast, Corro et al. (2017) determined that animals in herds of 200 or more were 1.5 times more likely to be infected than animals in smaller herds in Bolívar, Venezuela. Regarding Nc, it has been reported in several studies that the inadequate handling by the personnel in charge of the animals and the presence of rodents (Arauco 2018), the number of dogs in the herd, and the outdoor disposal of animal remains (Portocarrero et al. 2015) are risk factors for bovine neosporosis. However, these risk factors were beyond the scope of our study, and further research comprising such issues in the Department of Antioquia is needed.

## Conclusions

BLV seroprevalence in plasma samples from specialized dairy herds in Antioquia was 41.13%. The seroprevalence of Nc and BVD in the serum samples was 28.48% and 22.7%, respectively. Such high seroprevalences indicate the increased possibility of pathogen dissemination within and between the Department of Antioquia dairy herds and the possible effect of various aspects linked to the production system, such as management practices. A serological diagnosis of BLV, BVD, and Nc is proposed as diagnostic alternatives in dairy herds.

The presence of BLV was not detected in two herds, BVD in 15 herds, and Nc in seven herds, which shows that it is possible to have pathogen-free herds in specialized dairies in Antioquia. Given the high prevalence of these pathogens in other herds in the Department of Antioquia and Colombia, it should be noted that it may be an indicator of good management and sanitary control carried out in the herds where these samples come from.

High seroprevalence to BLV, BVD, and Nc was found in individuals with more than three parturitions. This indicates that, at the herd level, these animals may become the most affected in their productive and reproductive indices.

It is necessary to work on these issues more closely in the Department of Antioquia and Colombia, integrating producers, professionals (e.g., animal technicians and veterinarians), and livestock unions to raise awareness about the importance of adequate control of these pathogens. Additionally, all of these stakeholders must be taught about proper animal management, seeking to continue with research that contributes to improving milk production in Antioquia-Colombia.

## Data Availability

The datasets generated and/or analyzed during the current study are not publicly available because the information contains proper names of herds and producing areas participating in the study, but are available from the corresponding author upon reasonable request.

## Declarations

### Knowledge statements

The manuscript does not contain clinical studies or patient data.
